# Supplementary material for: Optimized customer churn prediction using tabular generative adversarial network (GAN)-based hybrid sampling method and cost-sensitive learning
Source: PeerJ Comput Sci. 2025 Jun 19;11:e2949. doi: 10.7717/peerj-cs.2949 (PMC12193428; doi:10.7717/peerj-cs.2949)
Supplement: Supplemental Information 1 [file peerj-cs-11-2949-s001.docx]

**DATASETS DATA DICTIONARY AND CODEBOOK**

**Telco 1**

<https://www.kaggle.com/datasets/blastchar/telco-customer-churn>

| **No** | **Feature** | **Description** |
| --- | --- | --- |
| 1 | CustomerID | Unique identifier for each customer. |
| 2 | Gender | Whether the customer is a male or a female. |
| 3 | SeniorCitizen | Whether the customer is a senior citizen or not (1 for Yes, 0 for No). |
| 4 | Partner | Whether the customer has a partner or not (Yes, No). |
| 5 | Dependents | Whether the customer has dependents or not (Yes, No). |
| 6 | Tenure | Number of months the customer has stayed with the company. |
| 7 | PhoneService | Whether the customer has a phone service or not (Yes, No). |
| 8 | MultipleLines | Whether the customer has multiple lines or not (Yes, No, No phone service). |
| 9 | InternetService | Customer’s internet service provider (DSL, Fiber optic, No). |
| 10 | OnlineSecurity | Whether the customer has online security or not (Yes, No, No internet service). |
| 11 | OnlineBackup | Whether the customer has online backup or not (Yes, No, No internet service). |
| 12 | DeviceProtection | Whether the customer has device protection or not (Yes, No, No internet service). |
| 13 | TechSupport | Whether the customer has tech support or not (Yes, No, No internet service). |
| 14 | StreamingTV | Whether the customer has streaming TV or not (Yes, No, No internet service). |
| 15 | StreamingMovies | Whether the customer has streaming movies or not (Yes, No, No internet service). |
| 16 | Contract | The contract term of the customer (Month-to-month, One year, Two year). |
| 17 | PaperlessBilling | Whether the customer has paperless billing or not (Yes, No). |
| 18 | PaymentMethod | The customer’s payment method (Electronic check, Mailed check, Bank transfer (automatic), Credit card (automatic)). |
| 19 | MonthlyCharges | The amount charged to the customer monthly. |
| 20 | TotalCharges | The total amount charged to the customer. |
| 21 | Churn | Whether the customer churned or not (Yes or No). |

**Bank**

<https://www.kaggle.com/datasets/shrutimechlearn/churn-modelling>

| No | **Feature** | **Description** |
| --- | --- | --- |
| 1 | CustomerId | Unique IDs for bank customer identification. |
| 2 | Surname | Customer's last name. |
| 3 | CreditScore | Credit score of the customer. |
| 4 | Geography | The country from which the customer belongs. |
| 5 | Gender | Male or Female. |
| 6 | Age | Age of the customer. |
| 7 | Tenure | Number of years for which the customer has been with the bank. |
| 8 | Balance | Bank balance of the customer. |
| 9 | NumOfProducts | Number of bank products the customer is utilizing. |
| 10 | HasCrCard | Whether the customer has a credit card (1 for Yes, 0 for No). |
| 11 | IsActiveMember | Whether the customer is an active member (1 for Yes, 0 for No). |
| 12 | EstimatedSalary | Estimated salary of the customer. |
| 13 | Exited | Whether the customer exited the bank (1 for Yes, 0 for No). |

**Mobile**

<https://www.kaggle.com/datasets/dimitaryanev/mobilechurndataxlsx>

| **No** | **Feature** | **Description** |
| --- | --- | --- |
| 1 | year | Year |
| 2 | month | Month |
| 3 | user_account_id | Unique customer identifier |
| 4 | user_account_id | Customer aging in months |
| 5 | user_intake | New customer identifier |
| 6 | user_no_outgoing_activity_in_days | Number of days when customer did not do any action |
| 7 | user_account_balance_last | Customer account balance at the end of the period |
| 8 | user_spendings | Revenue spend in the period |
| 9 | user_has_outgoing_calls | Customer made at least 1 call |
| 10 | user_has_outgoing_sms | Customer made at least 1 sms |
| 11 | user_use_gprs | Customer used data at least once |
| 12 | user_does_reload | Customer has done at least 1 recharge |
| 13 | reloads_inactive_days | Number of days without recharge |
| 14 | reloads_count | Number of recharges |
| 15 | reloads_sum | Amount of recharges |
| 16 | calls_outgoing_count | Number of outgoing calls |
| 17 | calls_outgoing_spendings | Amount spent on outgoing calls |
| 18 | calls_outgoing_duration | Duration of all outgoing calls |
| 19 | calls_outgoing_spendings_max | The most expensive call per period |
| 20 | calls_outgoing_duration_max | The longest call per period |
| 21 | calls_outgoing_inactive_days | Number of days without outgoing calls |
| 22 | calls_outgoing_to_onnet_count | Number of calls to on-net |
| 23 | calls_outgoing_to_onnet_spendings | Amount spent on outgoing calls to on-net |
| 24 | calls_outgoing_to_onnet_duration | Duration of all outgoing calls to on-net |
| 25 | calls_outgoing_to_onnet_inactive_days | Number of days without outgoing call to on-net |
| 26 | calls_outgoing_to_offnet_count | Number of calls to off-net |
| 27 | calls_outgoing_to_offnet_spendings | Amount spent on outgoing calls to off-net |
| 28 | calls_outgoing_to_offnet_duration | Duration of all outgoing calls to off-net |
| 29 | calls_outgoing_to_offnet_inactive_days | Number of days without outgoing call to off-net |
| 30 | calls_outgoing_to_abroad_count | Number of calls to other countries |
| 31 | calls_outgoing_to_abroad_spendings | Amount spent on outgoing calls to other countries |
| 32 | calls_outgoing_to_abroad_duration | Duration of all outgoing calls to other countries |
| 33 | calls_outgoing_to_abroad_inactive_days | Number of days without outgoing call to other countries |
| 34 | sms_outgoing_count | Number of outgoing sms messages |
| 35 | sms_outgoing_spendings | Amount spend on outgoing sms messages |
| 36 | sms_outgoing_spendings_max | The most expensive sms message |
| 37 | sms_outgoing_inactive_days | Number of days without outgoing sms message |
| 38 | sms_outgoing_to_onnet_count | Number of outgoing sms messages to on-net |
| 39 | sms_outgoing_to_onnet_spendings | Amount spend on outgoing sms messages to on-net |
| 40 | sms_outgoing_to_onnet_inactive_days | Number of days without outgoing sms message to on-net |
| 41 | sms_outgoing_to_offnet_count | Number of outgoing sms messages to off-net |
| 42 | sms_outgoing_to_offnet_spendings | Amount spend on outgoing sms messages to off-net |
| 43 | sms_outgoing_to_offnet_inactive_days | Number of days without outgoing sms message to off-net |
| 44 | sms_outgoing_to_abroad_count | Number of outgoing sms messages to other countries |
| 45 | sms_outgoing_to_abroad_spendings | Amount spend on outgoing sms messages to other countries |
| 46 | sms_outgoing_to_abroad_inactive_days | Number of days without outgoing sms message to other countries |
| 47 | sms_incoming_count | Number of incoming sms messages |
| 48 | sms_incoming_spendings | Amount spent on incoming sms messages |
| 49 | sms_incoming_from_abroad_count | Number of incoming sms messages from other countries |
| 50 | sms_incoming_from_abroad_spendings | Amount spend on incoming sms messages from other countries |
| 51 | gprs_session_count | Number of data connections |
| 52 | gprs_usage | Number of kb used |
| 53 | gprs_spendings | Money amount spent on data |
| 54 | gprs_inactive_days | Number of days without data usage |
| 55 | last_100_reloads_count | Number of recharges over the last 100 days |
| 56 | last_100_reloads_sum | Amount of recharges over the last 100 days |
| 57 | last_100_calls_outgoing_duration | Calls outgoing duration over the last 100 days |
| 58 | last_100_calls_outgoing_to_onnet_duration | Calls outgoing to on-net duration over last 100 days |
| 59 | last_100_calls_outgoing_to_offnet_duration | Calls outgoing to off-net duration over last 100 days |
| 60 | last_100_calls_outgoing_to_abroad_duration | Calls outgoing to other countries duration over last 100 days |
| 61 | last_100_sms_outgoing_count | Number of SMS messages over 100 days |
| 62 | last_100_sms_outgoing_to_onnet_count | Number of SMS messages to on-net over 100 days |
| 63 | last_100_sms_outgoing_to_offnet_count | Number of SMS messages to off-net over 100 days |
| 64 | last_100_sms_outgoing_to_abroad_count | Number of SMS messages to other countries over 100 days |
| 65 | last_100_gprs_usage | Number of kb used over last 100 days |

**Telco 2**

<https://www.kaggle.com/competitions/customer-churn-prediction-2020>

| No | **Feature** | **Description** |
| --- | --- | --- |
| 1 | state | String. 2-letter code of the US state of customer residence. |
| 2 | account_length | Numerical. Number of months the customer has been with the current telecom provider. |
| 3 | area_code | String. "area_code_AAA" where AAA is the 3-digit area code. |
| 4 | international_plan | (yes/no). Indicates if the customer has an international plan. |
| 5 | voice_mail_plan | (yes/no). Indicates if the customer has a voice mail plan. |
| 6 | number_vmail_messages | Numerical. Number of voice-mail messages. |
| 7 | total_day_minutes | Numerical. Total minutes of day calls. |
| 8 | total_day_calls | Numerical. Total number of day calls. |
| 9 | total_day_charge | Numerical. Total charge of day calls. |
| 10 | total_eve_minutes | Numerical. Total minutes of evening calls. |
| 11 | total_eve_calls | Numerical. Total number of evening calls. |
| 12 | total_eve_charge | Numerical. Total charge of evening calls. |
| 13 | total_night_minutes | Numerical. Total minutes of night calls. |
| 14 | total_night_calls | Numerical. Total number of night calls. |
| 15 | total_night_charge | Numerical. Total charge of night calls. |
| 16 | total_intl_minutes | Numerical. Total minutes of international calls. |
| 17 | total_intl_calls | Numerical. Total number of international calls. |
| 18 | total_intl_charge | Numerical. Total charge of international calls. |
| 19 | number_customer_service_calls | Numerical. Number of calls to customer service. |
| 20 | churn | (yes/no). Indicates whether the customer has churned. This is the target variable. |

We cannot provide the codebook for Telco 3 and insurance datasets, because it is not available on the original source

Here is the link source

<https://www.kaggle.com/datasets/royjafari/customer-churn>

<https://www.kaggle.com/datasets/k123vinod/insurance-churn-prediction-weekend-hackathon>
